# A Rare Case of a Morel-Lavallée Lesion Complicated by Pseudomonas aeruginosa Infection: Diagnostic Challenges and Therapeutic Considerations

**DOI:** 10.7759/cureus.48622

**Published:** 2023-11-10

**Authors:** Mohammad Yousaf, Rivers A Hock, Ethan Heh, Mark Raynor, Diego P Peralta

**Affiliations:** 1 Internal Medicine, Paul L. Foster School of Medicine, Texas Tech University Health Sciences Center El Paso, El Paso, USA; 2 Division of Infectious Diseases, Texas Tech University Health Sciences Center El Paso, El Paso, USA

**Keywords:** surgical and clinical management, soft tissue infection, pseudomonas aeruginosa, internal degloving injury, morel-lavallée lesion

## Abstract

Morel-Lavallée lesions are uncommon soft tissue injuries resulting from trauma, characterized by the separation of subcutaneous tissue from the underlying fascia. Soft tissue infections with *Pseudomonas aeruginosa* are rare and most typically associated with hospital-acquired infections and burn wounds. This case report is regarding a 57-year-old man following a motorcycle accident who presented with a unique occurrence of an MLL complicated by *P. aeruginosa* infection. The patient underwent extensive treatment over the course of months, which may have been prevented with a better understanding of the injury. This case is noteworthy due to the infrequency of the injury, the pathogen, and concomitant occurrence, presenting a diagnostic and therapeutic challenge. We describe the patient's clinical presentation, hospital course, diagnostic workup, and management to inform future care and recognition of similar patients.

## Introduction

A Morel-Lavallée lesion (MLL) is a closed injury with internal separation of superficial soft tissues from the deeper layers of fascia, also known as an internal degloving injury [1]. MLLs occur as shearing forces that cause separation of the superficial subcutaneous tissue from the underlying fascia, leaving a potential space for fluid collection and infection. In the acute phase, this lesion has swelling at the injury site. With time, the effusion becomes cystic and confined within the soft tissue and eventually, through an inflammatory response, may form a fibrous encapsulation [2]. These lesions often occur with high-energy blunt force trauma or crush injuries during motor vehicle collisions in regions such as thighs and are rarely recognized early [3]. These lesions are relatively rare; one trauma center identified 63 patients with MLLs within a ten-year time frame, while another noted 79 patients in a seven-year time frame [3,4]. The rarity of these lesions and the fact that they often occur in the setting of multiple distracting injuries contributes to them being frequently missed and, therefore, underdiagnosed [4]. There is no consensus regarding managing these lesions, but it can involve aspiration, sclerodesis, open debridement, and more conservative measures [5]. 

MLLs can give rise to soft tissue infection within the space created. Soft tissue infections can arise from a number of pathogens but are most often attributed to Staphylococcus and Streptococcus species [6]. The involvement of less common pathogens raises different diagnostic and therapeutic challenges. Among the various rare causative agents of soft tissue infection, Pseudomonas aeruginosa is particularly rare in the arena of soft tissue infections. It is characteristically seen in burn wounds, aquatic injuries, neutropenic patients, and hospital-acquired infections. It also has classic manifestations such as green nail syndrome, toe web infection, hot tub folliculitis, ear infections, and ecthyma gangrenosum [6,7]. As with many gram-negative bacteria, it has the potential for fatal disease. 

An MLL complicated by a P. aeruginosa infection is therefore an exceedingly unusual occurrence, especially in an immunocompetent patient such as in the present case. This case is presented not only to document this unusual event but also to highlight the challenges of diagnosing and managing such infections. Through this case report, we aim to contribute to understanding the clinical characteristics, diagnostic approach, and management considerations associated with MLLs complicated by P. aeruginosa infection. 

## Case presentation

Consent was obtained from the patient for the following images and clinical information for medical publication and presentation.

**Figure 1:**
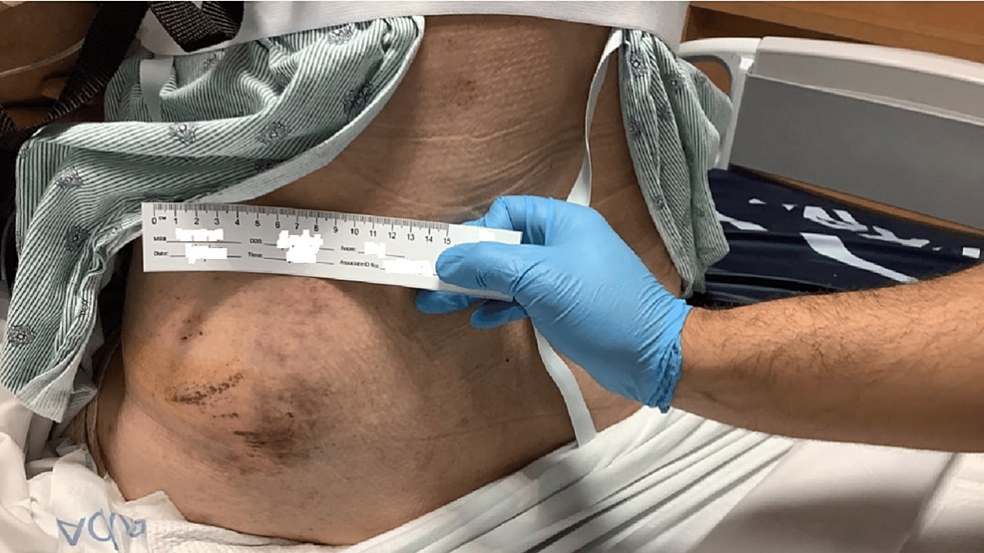
Images of the left lower extremity progression after surgical interventions January 2023: Large ecchymosis and road rash on the left lower back, hip, and lateral thigh following the motorcycle accident.

**Figure 2:**
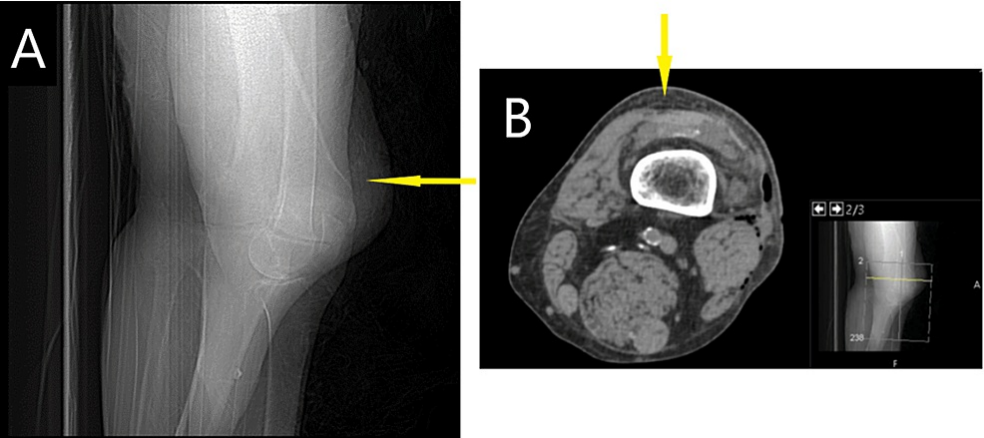
X-ray and CT scan without contrast of the left knee during the initial emergency department visit in January A) Initial X-ray showing a large amount of subcutaneous air with no evidence of acute bone or joint abnormalities. B) Initial CT showing broad anterolateral knee soft tissue defect with foci of superficial soft tissue gas.

This is the case of a 57-year-old man with a medical history of diabetes and obesity (BMI 32). He initially presented to the emergency department (ED) following a motorcycle accident in which he collided with a highway wall. His injuries included multiple rib fractures, a comminuted thumb fracture, blunt cardiac injury, avulsion of the soft tissue on the left anterior knee, ecchymosis, and road rash on the left hip and lateral thigh (Figure 1). During this initial visit, a knee CT scan and X-ray revealed signs consistent with a degloving injury of the left lateral thigh, though not an MLL at this time (Figure 2).

**Figure 3:**
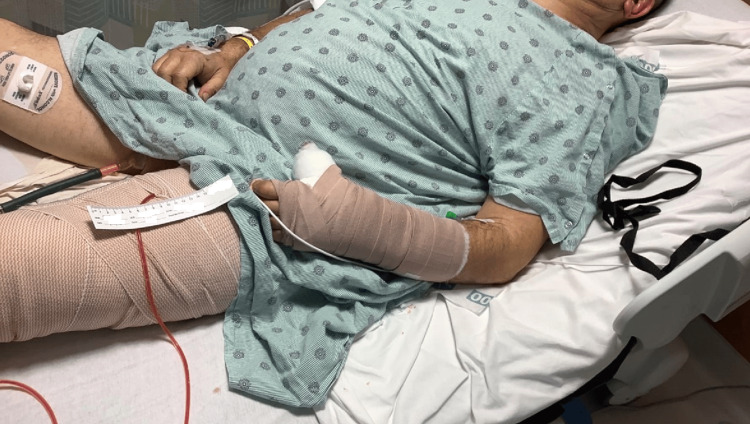
Images of the left lower extremity progression after surgical interventions Post-surgical dressings and drains placed on the left lower extremity after the first surgical intervention.

On the second day of admission, the patient underwent irrigation and debridement of the left lower extremity. During the procedure, sterile saline was injected into the knee capsule without evident fluid extravasation. The wound was irrigated and then loosely closed with 2-0 nylon. There was 270 cc of serosanguineous fluid output during the procedure, and dressings were placed along with a Hemovac drain and a Jackson-Pratt drain (Figure 3). He was discharged two weeks later to complete a 10-day course of cephalexin and to follow up in the clinic. Unfortunately, the patient did not attend the appointment.

**Figure 4:**
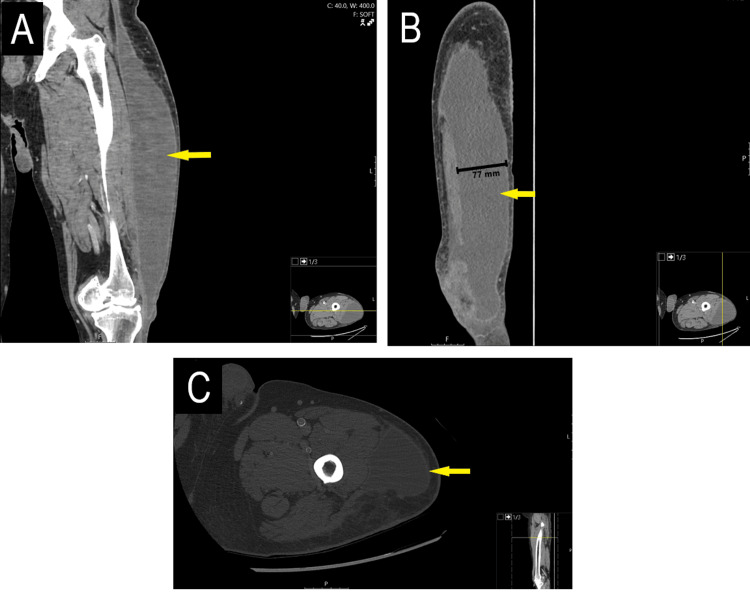
CT scan of the left lower extremity soft tissue with contrast during February hospitalization A) CT coronal lower bone with contrast showing the fluid collection. B) The collection measured 7.4 x 7.7 cm. C) Lower extremity soft tissue axial view showing collection of fluid. There is surrounding soft tissue edema in the fat of the left gluteal region.

About a month after his initial discharge, the patient returned to the ED due to increasing pain, fever, and chills at home. He reported completing the prescribed course of cephalexin. The physical examination revealed a large, soft, fluid-filled collection along the lateral thigh, extending to the knee, on the left leg. A follow-up CT scan of the left lower extremity showed a significant fluid collection in the deep soft tissues, extending along the lateral aspect of the fascia lata (Figure 4). These findings, along with the patient's history, were indicative of a MLL. The plastic surgery team was consulted for drainage and complete removal of the lesion's capsule.

During the procedure, a substantial fluid collection was observed extending down to the knee, aligned with the severe road rash. Approximately three liters of serosanguineous fluid were drained from the cavity. Further exploration revealed a mature capsule with a slick biofilm, which was excised along with a massive 53 cm x 6 cm portion of the lateral thigh. Following the capsulectomy, the area was thoroughly irrigated, and skin flaps were advanced and sutured closed. Two drains and two wound vacuums were placed with adequate suction, and the entire leg was wrapped in web rolls and Ace bandages. Serosanguineous fluid output was observed in both drains over the next few days. The patient was started on empiric piperacillin/tazobactam until culture results returned. Eventually, the culture showed the presence of P. aeruginosa, and the patient's treatment was transitioned to oral levofloxacin upon discharge. He was discharged with a three-week prescription of levofloxacin in early March. 

**Figure 5:**
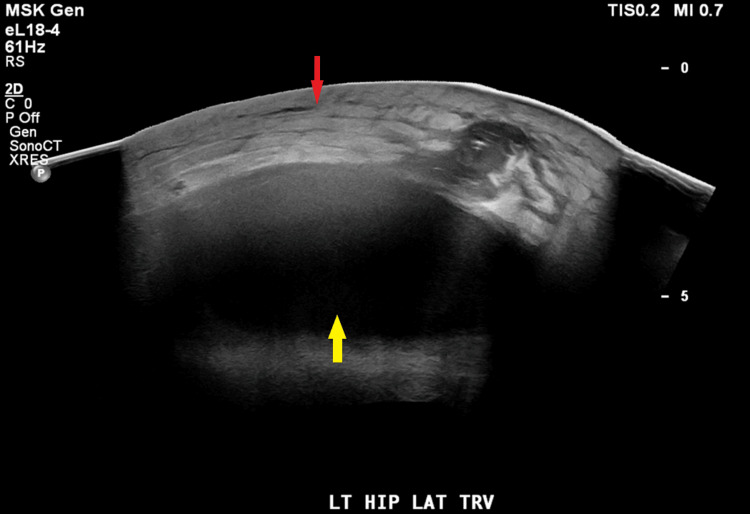
Ultrasound of left lower extremity taken during April hospitalization Ultrasound showed a large fluid collection (yellow) overlying the left hip/thigh with overlying soft tissue edema (red).

Over the next two months, the patient attended follow-up appointments with the plastic surgery team. He followed the recommended treatment plan and had both drains removed. However, four months after admission, the patient returned to the ED one week after drain removal due to increased pain and new fluid collection extending up to the left thigh. An ultrasound was obtained at this time due to concerns about DVT (Figure 5).

**Figure 6:**
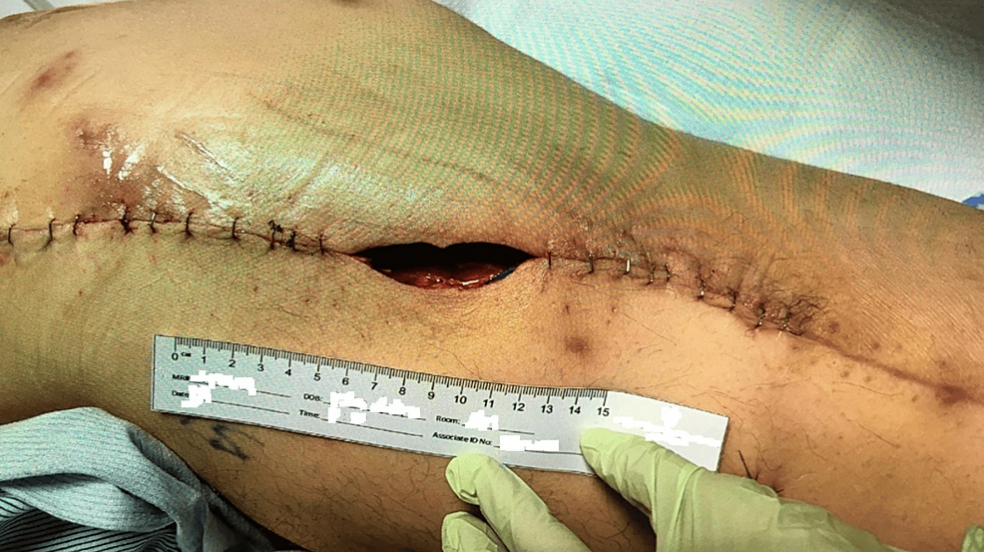
Images of the left lower extremity progression after surgical interventions April 2023: Post-surgical ten-centimeter open wound area left open for packing.

The plastic surgery team initiated a regimen of vancomycin and piperacillin/tazobactam and scheduled the patient for fluid collection drainage with culture. Around 500 cc of fluid was drained during the procedure, and a thorough debridement of the recurrent MLL with capsulectomy was performed. A 10-centimeter open wound area was purposely left open to be packed during wound care (Figure 6), and a wound vacuum was applied to obliterate the dead space. A Jackson-Pratt drain was left in place. The patient experienced post-operative anemia and acute kidney injury. Hence, he was continued only on empiric piperacillin/tazobactam. Culture results indicated mixed bacteria with methicillin-susceptible Staphylococcus aureus as the predominant organism. At this point, antibiotic therapy was adjusted to cefazolin and transitioned to oral cephalexin upon discharge. The susceptibilities of the S. aureus infection and the prior P. aeruginosa infection are shown in Table 1.

**Table 1:** Pseudomonas aeruginosa and Staphylococcus aureus susceptibility profile and interpretation S: Susceptible, I: Intermediate, R: Resistant MIC: Minimum Inhibitory Concentration

Pathogen	Pseudomonas aeruginosa		Staphylococcus aureus	
	Interpretation	MIC (µg/mL)	Interpretation	MIC (µg/mL)
Amikacin	S	<=8		
Cefepime	S	2		
Ceftaroline			S	0.25
Chloramphenicol			S	8
Clindamycin			R	<=0.5
Daptomycin			S	<=0.5
Doxycycline			S	<=0.5
Erythromycin			R	>4
Ciprofloxacin	S	<=0.25		
Gentamicin	S	<=2	S	<=1
Levofloxacin	S	1	S	<=1
Linezolid			S	<=1
Meropenem	S	<=0.5		
Minocycline			S	<=1
Moxifloxacin			S	<=0.5
Oxacillin			S	0.5
Penicillin			R	1
Piperacillin/Tazobactam	S	8/4		
Rifampin			S	<=0.25
Tetracycline			S	<=0.5
Tigecycline			S	<=0.125
Tobramycin	S	<=2		
Trimethoprim/Sulfamethoxazole			S	<=0.5/9.5
Vancomycin			S	1

**Figure 7:**
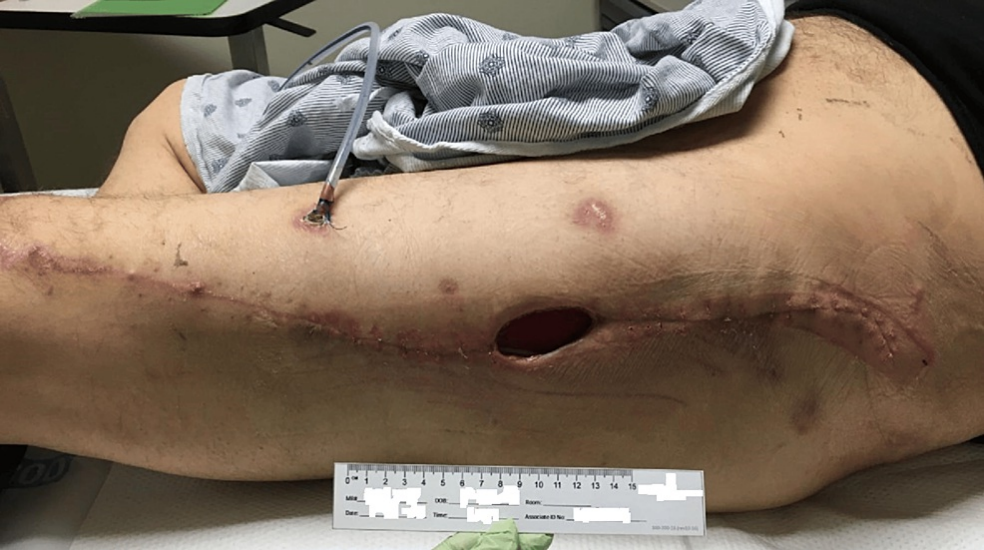
Images of the left lower extremity progression after surgical interventions May 2023: Smaller post-surgical wound with no signs of infection.

He was discharged with a four-week cephalexin prescription, and plans were made for interval CT scans to assess the need for continued therapy. One month after discharge, the wound had contracted to half its original volume with no signs of infection (Figure 7).

## Discussion

Based on an extensive literature search in the PubMed and Google Scholar databases, only four cases of MLLs with associated P. aeruginosa infection were found. The details of those four cases are included in Table 2.

**Table 2:** Morel-Lavallée lesion complicated by Pseudomonas aeruginosa infection reported cases in PubMed and Google Scholar databases

Author(s) and Year	Study Type	Age and Sex	Mechanism of Injury	Morel-Lavallée lesion Diagnosis	Management	Pathogen(s) Isolated	Outcome
Pikkel et al., 2020 [8]	Case report	59 years Male	Crushing injury to the right thigh with no fracture	Twelve days after the injury	Surgical drainage Two split-thickness skin grafts Ciprofloxacin and Cefazolin	Staphylococcus aureus Pseudomonas aeruginosa	Recovered
Shimizu et al., 2015 [9]	Case report	32 years Male	Car accident with unstable left pelvic fracture and fractures of lumbar transverse processes	Seven days after the injury	Transcatheter angiographic embolization Several debridements Left hemipelvectomy Broad-spectrum antibiotics not specified	Citrobacter species Enterobacter species Enterococcus species Pseudomonas aeruginosa	Recovered
Kim et al., 2015 [10]	Case series	53 years Male	Pedestrian traffic accident with pelvic bone fracture	Four days after the injury	Massive debridement Split-thickness skin graft and a local flap Intravenous antibiotics not specified	Pseudomonas aeruginosa	Recovered
Steiner et al., 2008 [11]	Case series	43 years Male	Car accident with type C pelvis injury and femur fracture	Within 24 hours of the injury	External fixator and intramedullary nailing Repeated debridements Wound VAC therapy Gentamicin and Clindamycin	Enterobacter cloacae Acinetobacter baumannii Pseudomonas aeruginosa	Recovered

Our case exemplifies the complexity of MLLs, which, even when recognized early, can lead to recurrence and complications. In many instances, MLLs remain undetected during the initial evaluation, particularly when they coexist with other injuries. Studies by Hudson et al. (1996) and others have shown that MLLs are frequently missed during the initial assessment. Hudson et al. (1996) in a single-center study reported that only one out of seven patients had their MLLs recognized on initial presentation following acute injury, while the remaining cases were initially overlooked [12]. Another study by Rodriguez-Roiz et al. (2022) involving nine patients found an average delay of 11.9 days before MLLs were identified after the initial injury [13]. Many of these patients had concurrent distracting injuries, likely contributing to the initial oversight. In such cases, MLLs are often mislabeled as seromas or hematomas.

While magnetic resonance imaging is a valuable tool for locating MLLs within tissue layers, our patient's case demonstrates that routine diagnostic methods such as CT scans and X-rays can also be instrumental in early detection, provided clinical suspicion remains high, though MRI remains the investigation of choice [1].

MLLs are challenging to diagnose early on, so it is important to identify situations that should trigger heightened clinical suspicion. A crucial factor is comprehending the mechanism of injury, as MLLs result from shearing forces that separate the superficial fascia from deeper fascial layers, necessitating a high-energy mechanism of injury such as a motor vehicle collision or sports-related trauma to generate such forces [5]. Consequently, MLLs often coincide with fractures. An analysis of 200 MLL cases revealed that the trochanteric/hip region was the most common site for MLL development, followed by the thigh, pelvis, knee, gluteal region, lumbosacral area, abdomen, calf/lower leg, and finally, the head [14]. Upon initial evaluation, patients may present with ecchymosis, soft tissue swelling, fluctuance, or skin hypermobility in the affected area, although these signs may manifest late in the clinical course [15]. Given the potential absence of these signs during the initial presentation, a thorough understanding of the injury mechanism remains invaluable for raising clinical suspicion and guiding the appropriate use of imaging studies. In this case, the patient had severe road rash on his thigh in the setting of a motorcycle accident, which raised suspicion for a shearing injury, and the cavity was able to be identified on imaging and subsequently drained.

The initial management of our patient involved irrigation and debridement, a crucial step in the successful treatment of MLLs. Effective closure of the space is essential, as failure to do so can lead to reaccumulation of fluid and expansion of the lesion. This technique may have been specially indicated in our patient due to the extensive tissue damage surrounding the lesion. An alternative and potentially more effective method of MLL treatment is sclerodesis using doxycycline. During this procedure, the lesion is aspirated and then infiltrated with a doxycycline solution, resulting in a reported resolution rate of 95.7% [1,16].

The post-operative management of MLLs involves several key factors aimed at preventing recurrence. It includes vigilant drainage monitoring and drain removal once output falls below 30 mL/day [5]. The literature consistently emphasizes the importance of post-operative compression, with its absence often leading to treatment failure [5,17]. It is crucial to convey these factors clearly to patients to minimize the risk of recurrence and complications, such as infection.

In our case, the emergence of a unique P. aeruginosa infection added to the complexity of the recurring MLL, a phenomenon rarely reported in the literature. Pathogens such as P. aeruginosa, S. aureus, Candida, and mycobacteria can produce biofilms. These biofilms are very difficult or impossible to eradicate unless physically removed. In this patient, surgical debridement and capsule removal were done to eradicate biofilms. Studies have shown antibiotic combinations such as gentamicin/ciprofloxacin and tobramycin/clarithromycin to have efficacy against P. aeruginosa biofilms [18]. Other current strategies include using dornase alfa alongside antibiotics to enhance biofilm removal and the use of nitric oxide or cephalosporin-3’-diazeniumdiolates to mediate biofilm dispersal. However, these and others are less commonly implemented and have limited data supporting their efficacy in vivo [19]. The resistance these biofilms provide and the difficulty in removing them make early diagnosis and treatment before their formation very important. It underscores the significance of empiric antibiotic treatment, strict post-operative adherence, and frequent follow-up. A recurrence or delayed diagnosis of MLL elevates the risk of capsule formation, as observed in this case. Capsule formation necessitates surgical intervention for removal, as it carries the highest risk of continued MLL recurrence [5].

## Conclusions

Our case study shows that MLLs can be complex and challenging to manage. They can recur and have complications, so it is essential to recognize them early and treat them promptly. It includes meticulous surgical management and post-operative care. The occurrence of a P. aeruginosa infection in our case highlights the need for comprehensive antibiotic treatment, patient education, and vigilance with follow-up to achieve the best outcomes.
